# Chemotactic synthetic vesicles: Design and applications in blood-brain barrier crossing

**DOI:** 10.1126/sciadv.1700362

**Published:** 2017-08-02

**Authors:** Adrian Joseph, Claudia Contini, Denis Cecchin, Sophie Nyberg, Lorena Ruiz-Perez, Jens Gaitzsch, Gavin Fullstone, Xiaohe Tian, Juzaili Azizi, Jane Preston, Giorgio Volpe, Giuseppe Battaglia

**Affiliations:** 1 Department of Chemistry, University College London, 20 Gordon Street, London WC1H 0AJ, UK.; 2 Sunnybrook Research Institute, 2075 Bayview Avenue, Toronto, Ontario M4N 3M5, Canada.; 3 Department of Chemistry, University of Basel, Klingelbergstrasse 80, 4056 Basel, Switzerland.; 4 Institute for Cell Biology and Immunology, University of Stuttgart, Allmandring 31, Stuttgart 70569, Germany.; 5 School of Life Sciences, Anhui University, Hefei 230039, PR China.; 6 Institute of Pharmaceutical Science, Kings College London, 150 Stamford Street, London SE1 9NH, UK.; 7 Department of Chemical Engineering, University College London, Torrington Place, London WC1E 7JE, UK.

## Abstract

In recent years, scientists have created artificial microscopic and nanoscopic self-propelling particles, often referred to as nano- or microswimmers, capable of mimicking biological locomotion and taxis. This active diffusion enables the engineering of complex operations that so far have not been possible at the micro- and nanoscale. One of the most promising tasks is the ability to engineer nanocarriers that can autonomously navigate within tissues and organs, accessing nearly every site of the human body guided by endogenous chemical gradients. We report a fully synthetic, organic, nanoscopic system that exhibits attractive chemotaxis driven by enzymatic conversion of glucose. We achieve this by encapsulating glucose oxidase alone or in combination with catalase into nanoscopic and biocompatible asymmetric polymer vesicles (known as polymersomes). We show that these vesicles self-propel in response to an external gradient of glucose by inducing a slip velocity on their surface, which makes them move in an extremely sensitive way toward higher-concentration regions. We finally demonstrate that the chemotactic behavior of these nanoswimmers, in combination with LRP-1 (low-density lipoprotein receptor–related protein 1) targeting, enables a fourfold increase in penetration to the brain compared to nonchemotactic systems.

## Teaser

Brain homing nanoswimmers: Glucose-fueled propulsion combined with blood-brain barrier crossing enhances brain delivery.

## INTRODUCTION

Directional locomotion or taxis is possibly one of the most important evolutionary milestones, because it has enabled many living organisms to outperform their nonmotile competitors. In particular, chemotaxis (that is, the movement of organisms either toward or away from specific chemicals) (*1*, *2*) is possibly the most common strategy adopted by many unicellular organisms to gather nutrients, escape toxins (*3*), and help coordinate collective behaviors such as the formation of colonies and biofilms (*4*). Chemotaxis is also exploited by multicellular systems for tissue development (*5*), immune responses (*6*), or cancer metastasis (*7*). It enables long-range interactions that extend over length scales that are several orders of magnitude larger than the motile system itself (*8*). It is not surprising that scientists have been trying to design devices that mimic such a behavior (*9*–*12*). When swimming is scaled down to the microscale, the fluid dynamics are dominated by viscous rather than inertial forces (that is, Stokes regime). Under these conditions, propulsion is possible only by not-time-reversible deformations of the swimmer’s body (*13*, *14*) or by inducing a phoretic slip velocity on the swimmer’s surface (*15*, *16*). The latter can, for example, be achieved by creating thermal gradients (thermophoresis) or chemical gradients of either charged (electrophoresis) or neutral (diffusiophoresis) solutes in the swimmer’s environment (*15*). Recently, it has in fact been proposed that the swimmer can induce a slip velocity on its surface by generating an asymmetric distribution of reaction products that creates a localized chemical gradient. This concept known as self-diffusiophoresis was formalized theoretically (*17*) and demonstrated experimentally using latex particles (*18*) and gold/silver rods (*19*).

From a biotechnological point of view, self-propulsion can be applied to create carriers that are able to autonomously navigate within biological fluids and environments. This could enable directed access to nearly every site of the human body through blood vessels, independent of the blood flow and local tissue architectures. In this respect, recent preliminary experiments were performed with inorganic microparticles propelled by pH in the stomach of living mice (*20*). The ability to control active diffusion as a function of a physiological stimulus bodes well for tackling challenges in drug delivery where an efficient approach is yet to be found. Among these, the ability to deliver drugs within the central nervous system (CNS) is one of the most difficult tasks where current approaches only enable a small percentage of the injected dose to reach the brain and the spinal cord (*21*, *22*). The brain and the rest of the CNS are well guarded by physiological barriers, with the blood-brain barrier (BBB) being the most important. The BBB has the dual function to protect the CNS and to ensure that it receives an enhanced supply of metabolites. The brain is indeed the most expensive organ in our body (*23*), consuming almost 20% of oxygen and glucose. The latter is possibly one of the most important CNS nutrients (*24*), and the BBB regulates its passage very effectively, with a consequent high flow of glucose from the blood to the brain.

Here, we propose the design of an autonomous nanoscopic swimmer based on the combination of naturally occurring enzymes with fully biocompatible carriers, known as polymersomes, that have already been proven to hold great promise as drug and gene delivery vehicles (*25*, *26*). Specifically, to target the BBB and enter the CNS (*27*), we equip polymersomes with the ability to self-propel in the presence of glucose gradients.

## RESULTS AND DISCUSSION

### Asymmetric polymersomes

**Fig. 1:**
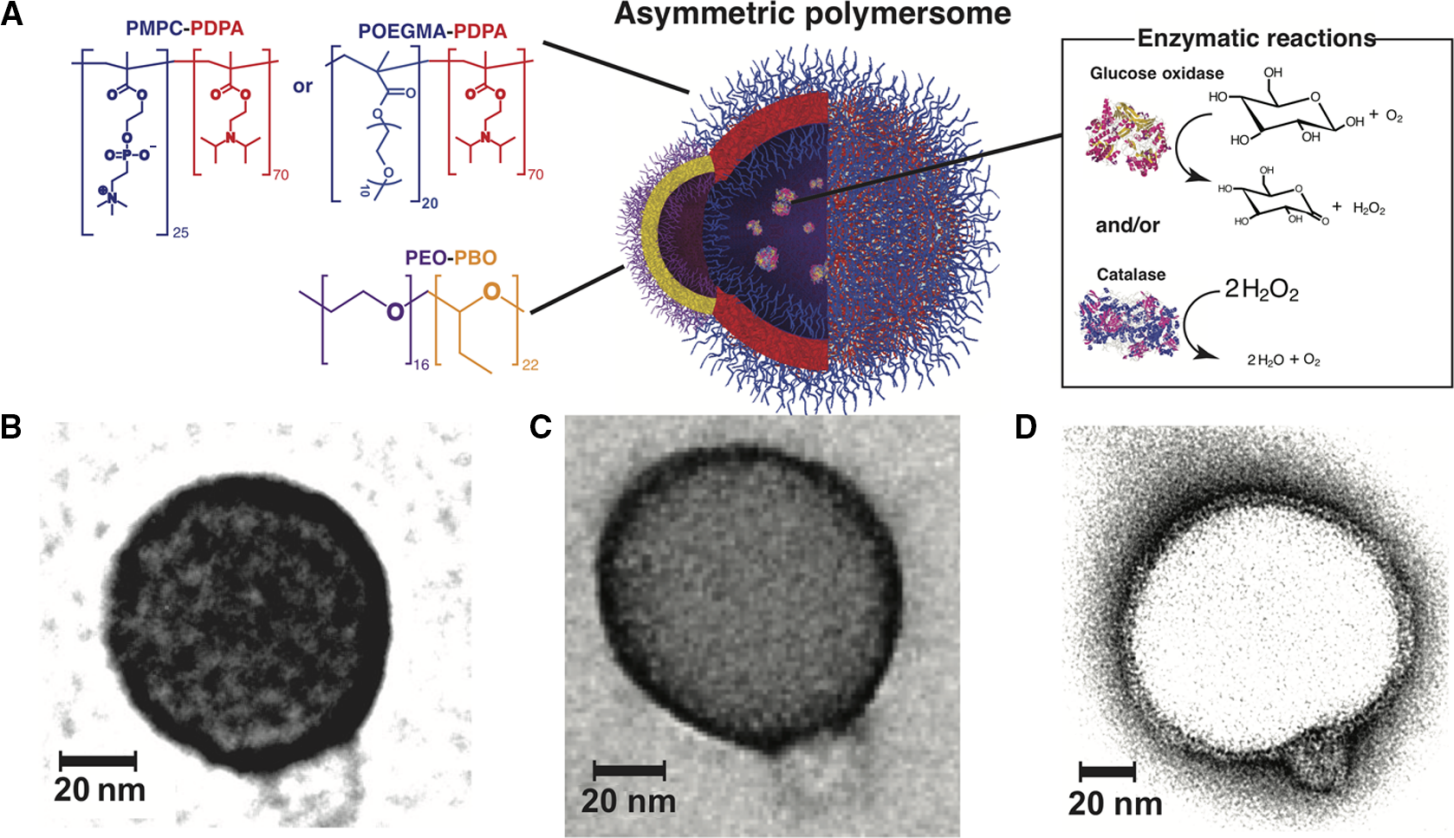
Asymmetric polymersomes. (**A**) Schematic representation of a chemotactic polymersome using a combination of membrane topology formed by PEO-PBO copolymers mixed with either PMPC-PDPA or POEGMA-PDPA copolymers. The polymersomes encapsulate glucose oxidase and/or catalase enzymes. (**B**) 9:1 PMPC-PDPA/PEO-PBO polymersome imaged in positive staining exploiting the high affinity of PDPA with the staining agent phosphotungstic acid (PTA). (**C**) 9:1 POEGMA-PDPA/PEO-PBO polymersome imaged in the same staining agent for PDPA. (**D**) 9:1 PMPC-PDPA/PEO-PBO polymersome imaged in negative staining to highlight the differences in membrane thickness between the PDPA and the PBO membrane.

Polymersomes are vesicles formed by the self-assembly of amphiphilic copolymers in water (*28*). They have been proposed as an alternative to liposomes (vesicles formed by naturally occurring phospholipids) because they offer greater flexibility over chemical and physical properties and allow large amounts of biological molecules, alone and in combination, including proteins and nucleic acids, to be compartmentalized into nanoscale reactors (*29*, *30*). Furthermore, we have demonstrated (*31*–*34*) that, when two different copolymers are used to form one polymersome, the resulting membrane segregates laterally into patterns whose topology is strictly controlled by the molar ratio of the two copolymers and eventually coarsen into two separate domains forming asymmetric polymersomes (*35*). Here, we exploit this asymmetry to achieve propulsion at the nanoscale. We mixed either poly[(2-methacryloyl)ethyl phosphorylcholine]–poly[2-(diisopropylamino)ethyl methacrylate] (PMPC-PDPA) or poly[oligo(ethylene glycol) methyl methacrylate] (POEGMA)–PDPA with poly(ethylene oxide) poly(butylene oxide) (PEO-PBO) copolymers. The copolymers were selected on three different complementary properties: (i) protein resistance for the hydrophilic blocks PEO, POEGMA, and PMPC to hinder unspecific interaction with plasma proteins (opsonization) and limit rapid riddance from the immune system; (ii) pH sensitivity for the PDPA to allow endosome escape and intracellular delivery; and (iii) high permeability for the PBO to preferentially channel both enzyme substrate and product diffusion. PMPC-PDPA and POEGMA-PDPA have been established in vivo (*36*, *37*), and whereas the PMPC can be used directly to target scavenger receptor B overexpressed in cancer cells (*38*), the POEGMA is inert in biological fluids and allows easy conjugation to decorate polymersome with ligands (*27*, *39*) to target specific cells. More relevantly, we show here that we can use POEGMA polymersomes as a platform for crossing the BBB and entering the CNS when combined with the low-density lipoprotein receptor–related protein 1 (LRP-1) targeting peptide Angiopep-2 (LA) (*27*). PEO-PBO forms very thin membranes (~2.4 nm) (*40*) that are highly permeable to most small polar molecules, such as hydrogen peroxide and glucose (*41*). The schematic of our proposed design is shown in Fig. 1A. We have previously demonstrated (*32*) that the two copolymers form asymmetric polymersomes at an optimal 9:1 molar ratio with the small permeable bud being formed by the minor PEO-PBO component. This can be verified using transmission electron microscopy (TEM) by imaging the polymersomes using positive staining selective for the PDPA blocks [see Fig. 1 (B and C) for the PMPC-PDPA/PEO-PBO and the POEGMA-PDPA/PEO-PBO mixtures]. As shown using negative staining TEM (Fig. 1D) where the PBO domain is darker, the thickness of the two membranes can be measured to be about 6.4 and 2.4 nm (for the PMPC-PDPA and PEO-PBO domains respectively), confirming previously reported measurements (*25*, *40*). We have already demonstrated that PBO membranes are 10 times more permeable than phospholipid ones (*41*) and that these are at least 10 times less permeable than thick membranes formed by aliphatic chains such as the PDPA ones (*42*). To a first approximation, we can thus infer that the PBO membrane is two orders of magnitude less permeable than the PDPA membrane. We can use such an asymmetric polymersome to encapsulate enzymes using a technique based on electroporation (*30*). We chose glucose oxidase to catalyze the glucose oxidation to form d-glucono-δ-lactone and hydrogen peroxide and catalase to catalyze the decomposition of hydrogen peroxide into water and oxygen. Both enzymes and reagents are naturally occurring in the human body. As shown in fig. S1, we encapsulated an average of six glucose oxidases and two catalases per polymersome either alone or in combination. We thus hypothesize that, as the enzymes react with their respective substrates, the confined reactions will produce a flux of products that will be preferentially expelled out of the polymersomes from the most permeable patch, that is, the bud formed by the minor PEO-PBO component. This in turn generates a localized gradient of the products that should set up the conditions for self-propulsion. The nature of the propulsion mechanism depends on the interaction between the reaction products and the two different polymersome domains (*15*). To a first approximation, this should set the conditions for self-diffusiophoresis where the depletion of the product molecules near the polymersome surface induces a lateral water flow with slip velocity **v**_S_. Assuming a spherical geometry of radius *R*, the polymersome propulsion translation and angular velocity can be derived from the slip velocity as **U** = − 1/*A*∮_*A*_**v_S_***dA* and Ω = 3/2*RA*∮_*A*_(**v_S_** × **n**)*dA*, respectively, where *A* is the total polymersome surface area and **n** is the polymersome orientation unit vector. This vector originates from the polymersome center of mass and is directed toward the center of the asymmetric PEO-PBO domain. Both velocities can be used to derive the general equations of motion expressed as a function of the polymersome position **r** and orientation unit vector **n** as

$$\frac{\partial r}{\partial t}=U+\sqrt{\frac{k_{B}T}{3\pi \eta R}}W_{t}(t)$$ (1)

$$\frac{\partial n}{\partial t}=\Omega \times n+\sqrt{\frac{k_{B}T}{4\pi \eta R^{3}}}W_{r}(t)\times n$$ (2)

where *k*_B_ is the Boltzmann constant, *T* is the absolute temperature, η is the water viscosity, **W**_t_ and **W**_r_ are the white noise vectors that respectively model the translational and rotational Brownian diffusion of the particle (*15*, *43*).

### Active diffusion analysis

To characterize the motility of the polymersomes, we have used a technique known as nanoparticle tracking analysis (NTA) (*44*). This is based on the dark-field parallel tracking of thousands of single nanoparticles using a camera to detect the light of a monochromatic laser scattered by the particles. The geometry of the observation chamber is shown in fig. S2, and unless specified differently, we performed all the measurements under physiological conditions, that is, *T* = 37°C, η_water_ = 0.69 mPa, pH 7.4, in 100 mM phosphate-buffered saline (PBS). The trajectories and the corresponding mean square displacements (MSDs) can be used to evaluate the motility of the polymersomes. In figs. S3 to S13, we show 1-s trajectories (all normalized to a common origin) and the corresponding MSDs for thousands of polymersomes imaged at 30 frames per second (fps) under different environmental conditions. In a homogeneous environment, either in the presence or absence of the substrate, the results show that, independently of being symmetric or asymmetric (loaded with enzymes or empty), the polymersomes have a typical Fickian diffusion profile with linear MSDs and stochastic trajectories. Although the MSDs averaged over thousands of trajectories (figs. S3 to S7) show some variations in the long-time diffusion coefficient, these variations are mainly due to statistical fluctuations between different experimental realizations of the process. In particular, we do not observe any appreciable enhancement in diffusivity. This suggests that even if the enzymatic reaction creates an asymmetric distribution of products around the loaded patchy polymersomes, with consequent propulsion velocity, any corresponding directed part of the motion is not sufficient to overcome the polymersome high rotational diffusion due to its small size [*z*-average measured by dynamic light scattering (DLS) *R* = 50 ± 10 nm; fig. S1], which effectively hinders any self-propulsion by effectively randomizing the particles’ orientations in τ ≈ 0.5 ms, one order of magnitude below our experimental time resolution (about 33.3 ms). To further confirm this, we calculated the ratio between the enhanced diffusion coefficient *D*_eff_ and the Stokes-Einstein diffusion coefficient *D*_0_ from two-dimensional (2D) projections of 3D-simulated trajectories of chemotactic polymersomes. If, in first approximation, we assume that Ω = 0, for a polymersome moving with a propulsion velocity of 100 μm s^−1^, *D*_eff_ = 1.15 *D*_0_, and for a polymersome moving at 200 μm s^−1^, the *D*_eff_ = 1.45 *D*_0_. A detectable enhancement of *D*_eff_ = 2*D*_0_ corresponds to a propulsion velocity of 300 μm s^−1^. These calculations confirm that any enhancement in diffusion is small for realistic values of size and velocity in our system, thus making it difficult to detect given the experimental variability. Both experiments and simulations therefore suggest that the angular phoretic term proportional to Ω in Eq. 2 is considerably smaller than the Brownian rotational component and hence can be ignored hereafter.

**Fig. 2:**
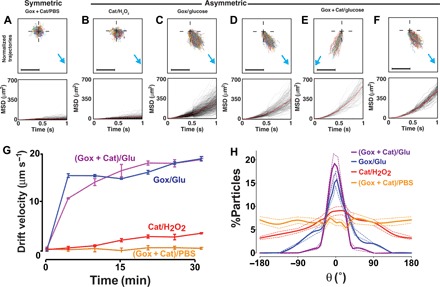
Active diffusion studies. Normalized 1s-trajectories and corresponding MSDs for (**A**) symmetric PMPC-PDPA polymersomes loaded with glucose oxidase (Gox) and catalase (Cat) and responding to a glucose gradient (**B**) asymmetric PMPC-PDPA/PEO-PBO polymersomes loaded with catalase and responding to a hydrogen peroxide gradient, (**C**) loaded with glucose oxidase and responding to a glucose gradient, (**D** to **E**) loaded with glucose oxidase and catalase responding to a glucose gradient coming (D) from the right-hand side and (E) from the left-hand side and for (**F**) asymmetric POEGMA-PDPA/PEO-PBO polymersomes loaded with glucose oxidase and catalase responding to a glucose gradient coming from the right-hand side. Blue arrows indicate the direction of the substrate gradient. Scale bars, 20 μm. (**G**) The average drift velocity is plotted as a function of time after the substrate addition for the previous experiments. The error bars represents the SE calculated over *n* = 3 measurements. (**H**) Degree of polarization of the corresponding trajectories towards the chemical gradient plotted as percentage of particles versus the gradient angle. Perfect alignment with the gradient corresponds to θ = 0°. The dashed lines represent the SEs.

**Fig. 3:**
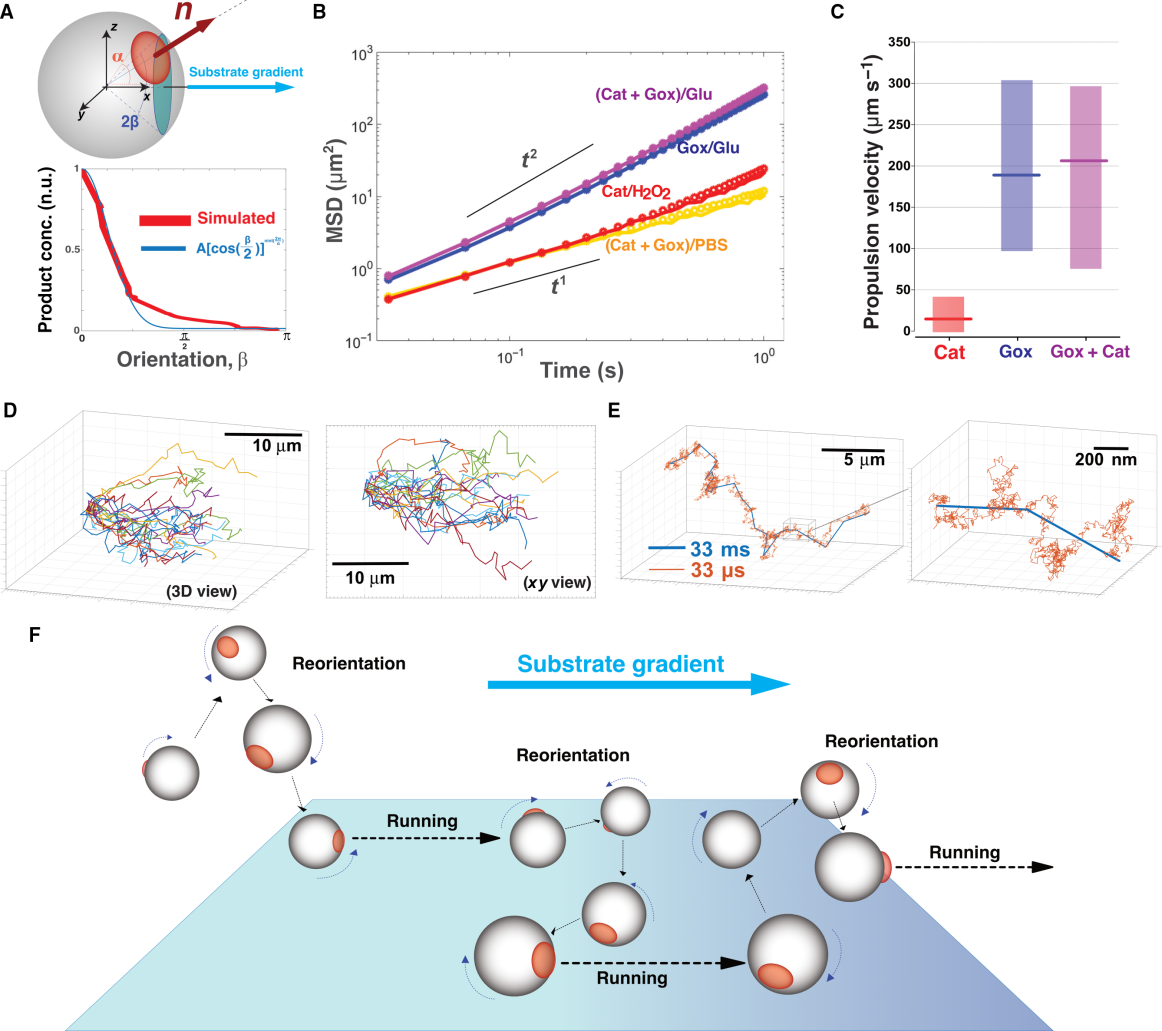
Mechanism of chemotaxis. (**A**) Schematics of an asymmetric polymersome and its reference axis. We assumed the polymersome to be a sphere (*R* = 50 nm) with a smaller patch (*r* = 15 nm and sector angle α); the angle β represents the orientation of the unit vector **n** with respect to the chemical gradient ∇**C** here aligned to the *x* axis. We simulated the distribution of the products around the polymersome, and their normalized concentration is plotted (red line) alongside a fitting function Δ*C*_p_ = *A*(cos(β/2))^nint(2π/α)^ (blue line). n.u., normalized units. (**B**) Average MSDs for both experimental (circles) and simulated data (solid line) for asymmetric polymersomes loaded with Gox and Cat responding to a glucose gradient (purple line and data) or in PBS (orange line and data), loaded with Gox and responding to a glucose gradient (blue line and data), and loaded with Cat responding to a hydrogen peroxide gradient (red line and data). (**C**) Corresponding propulsion velocities calculated by the numerical fittings for the three different combinations of enzymes and substrates. The lines represent the average values, whereas the bars represent the range of minimum and maximum calculated velocity in the sample. (**D**) A total of 20 simulated trajectories of Gox + Cat–loaded polymersomes using the same temporal steps as in the experiments (30 fps). These are shown as a 3D axonometric projection view and in the corresponding *xy* plane to show the comparison with the experimental data. (**E**) A single simulated 3D trajectory shown with temporal steps of 33 ms (blue line) and 33 μs (orange line). The detail of a single trajectory is zoomed to show the succession of reorientation and running steps of the polymersome diffusion. (**F**) Schematics of the proposed mechanisms of asymmetric polymersome chemotaxis, which consists of an alternation of running and reorientation events.

We repeated the same set of experiments described in figs. S3 to S7 in the presence of a concentration gradient created by adding the substrate from one side of the observation chamber (Fig. 2 and figs. S8 to S16). Under the new experimental conditions, the symmetric polymersomes (either loaded or empty) as well as the empty asymmetric polymersomes still showed a typical Fickian diffusion profile with stochastic trajectories and linear MSDs as a function of time. As a reference, Fig. 2A only shows the data corresponding to the case of symmetric polymersomes loaded with both glucose oxidase and catalase responding to a gradient of glucose (generated by a 1 M solution at the injection site), whereas the other control measurements are reported in figs. S3 to S16. The enzyme-loaded asymmetric polymersomes instead responded quite differently to the gradient of their respective substrate (Fig. 2, B to E). Figure 2B shows the data for the asymmetric polymersomes loaded with catalase alone (Cat) responding to a hydrogen peroxide gradient (generated by a 1 mM solution) coming from the right-hand side of the observation chamber; the normalized trajectories are biased toward the gradient, and the corresponding MSDs show a ballistic behavior with a quadratic dependence on time. We limited our experiments to low concentration of hydrogen peroxide to avoid its spontaneous decomposition and consequent formation of oxygen bubbles that could dissolve the polymersomes, as shown recently by Jang *et al*. (*45*). Such superdiffusive behavior is considerably more pronounced for the asymmetric polymersomes loaded either with glucose oxidase alone (Gox) (Fig. 2C) or glucose oxidase and catalase (Gox + Cat) together (Fig. 2, D and E), responding to a glucose gradient generated by a 1 M solution; almost all the trajectories are aligned toward the gradient, whether this comes from the right-hand (Fig. 2D) or the left-hand (Fig. 2E) side. This does not change when, instead of using PMPC-PDPA polymersomes, we use POEGMA-PDPA polymersomes, demonstrating that the differential permeability of PDPA and PBO is responsible for the self-phoresis (Fig. 2F). In addition to the trajectory and MSD analysis, the average drift velocities are plotted in Fig. 2G as a function of the time of observation after the substrate addition. For Brownian particles such as those in the control samples, the average drift velocity is zero, but as the samples become more chemotactic, the drift velocity gradually increases. The variation in drift velocity as a function of time after the addition of the substrate allows us to estimate how self-propulsion behavior varies with chemical gradient magnitude, and, in all cases, the drift velocity equilibrates to a plateau value corresponding to the time when the gradient becomes linear (that is, ∇*C* ≈ constant) and the system reaches steady-state conditions. This suggests that the rate of glucose diffusion matches the time scale of propulsion. Finally, the distribution of particle orientation with respect to the direction of the substrate gradient is plotted in Fig. 2H for all cases. Brownian samples (such as all the controls) have directions almost equally distributed across all angles, whereas, as the sample starts to exhibit propulsion and chemotaxis, the distribution of particles polarizes toward the direction of the gradient. All the data displayed in Fig. 2 show that, independently of the enzyme/substrate system, asymmetric polymersomes show typical ballistic behavior with a chemotactic response toward the enzyme substrate gradient marked hereafter as θ = 0. The catalase-loaded polymersomes respond rather weakly to the hydrogen peroxide gradient, and this is independent of the peroxide initial tested concentrations. Glucose oxidase–loaded asymmetric polymersomes, on the other hand, respond very strongly to a glucose gradient, reaching drift velocities around 20 μm s^−1^ with most particles polarized toward the gradient. Similar values are comparable to those of chemotactic bacteria, such as *Escherichia* *coli*, which are one order of magnitude larger than the polymersomes studied herein (*4*). As shown in Fig. 1A, glucose oxidase and catalase operate very well together because their respective reactions feed each other, with hydrogen peroxide being a product of glucose dissociation and the oxygen being a product of hydrogen peroxide dissociation (*46*). Furthermore, their combination leads to the formation of nondetrimental molecules because both oxygen and hydrogen peroxide are consumed and transformed into water and d-glucono-δ-lactone. Glucose oxidase self-regulates, and, as a critical concentration of hydrogen peroxide is reached, its activity is inhibited. This means that even at low H_2_O_2_ concentrations, we can assume that catalase consumes most of the H_2_O_2_ (*47*). Most notably, glucose oxidase– and catalase-loaded asymmetric polymersomes had the strongest response to glucose gradients and produced slightly higher drift velocities and considerably more polarized chemotaxis than the system loaded with glucose oxidase or catalase alone. From these data, we can conclude that no osmotic flow is generated, as demonstrated by all control measurements in the supplementary figures and that (i) the asymmetric distribution is critical, indeed symmetric polymersomes (either made of PDPA or PBO membranes) loaded with enzymes did not show any chemotactic drift; (ii) the reaction is critical, and empty polymersomes either symmetric or asymmetric do not exhibit any diffusophoretic drift due only to the substrate gradient; and, finally, (iii) only when the enzymes are encapsulated within an asymmetric polymersome is chemotaxis exhibited, suggesting that the propulsion velocity is only proportional to the products ∇*C*_p_. These conclusions suggest that, in the presence of a gradient, the strength of the polymersomes’ propulsion velocity is strongly biased by its orientation so as to create an asymmetric angular probability in the particle’s motion that is higher when the particle is oriented toward the gradient. The data in Fig. 2 are the 2D projections of 3D trajectories on the field-of-view plane. To simulate the same arrangement, we use a spherical polymersome with *R* = 50 nm and a smaller semispherical patch radius *r* = 15 nm, as shown in Fig. 3A. We assume that the chemical gradient is aligned along the *x* axis and that the orientation of the unit vector **n** is defined by a cone within the sphere with aperture 2β. We can simulate the distribution of the products’ concentration just outside the PBO permeable patch at different orientations β (note S2.4.2), and this is expectedly biased toward the chemical gradient as shown by the red line in Fig. 3A. We approximated this distribution with the function Δ*C*_p_ = *A*(cos(β/2))^nint(2π/α)^, where *A* is a proportionality constant and α is the sector angle of the PBO domain. Because the gradient in the product distribution around the particle is in first approximation proportional to such Δ*C*_p_ (*17*, *18*), the propulsion velocity can be estimated from the data by describing its functional form with the same modulation in the polymersome orientation. Such an approximation, together with the assumption that the polymersome’s phoretic angular velocity Ω is negligible when compared to its rotational diffusion, allows us to simulate the propulsion of the polymersomes in the presence of the substrate gradient by using Eqs. 1 and 2 (note S3). As shown in Fig. 3B, by fitting (solid lines) the experimental data (circles) with our model, we were able to estimate the strength of the propulsion velocity for each formulation (Fig. 3C). The (Gox + Cat) formulation is the one with the highest propulsion velocity, and the formulation with catalase alone in the presence of hydrogen peroxide is the one with the lowest value. The difference in performance of the two enzymes/substrates is possibly due to the difference in substrate concentration (considerably lower for the peroxide), which leads to a shallower gradient of products. Notably, we observe chemotaxis in all the different combinations, proving that the system we propose here works with very different combinations of substrate/enzyme. The simulations allow us to access the dynamics of propulsion with no limits in both spatial and temporal resolution. In Fig. 3D, we show the simulated 3D trajectories normalized to a common origin of 20 polymersomes with a temporal sampling identical to our experimental setting (that is, 33 ms corresponding to a 30-fps acquisition rate). We show these both as a 3D axonometric projection and in the corresponding *xy* plane view, which reproduce very closely the experimental data in Fig. 2. In Fig. 3E, a single trajectory is plotted using both temporal resolution of 33 ms (blue line) and 33 μs (orange line) corresponding to a 30 × 10^5^ and 3 × 10^5^ fps acquisition rate, respectively. The polymersome trajectories reveal that they are the result of a succession of running and reorientation events within the millisecond time scale, and hence, the polymersomes quickly reorient toward the gradient with consequent self-propulsion, as schematically represented in Fig. 3F.

### Chemotaxis in complex environments

**Fig. 4:**
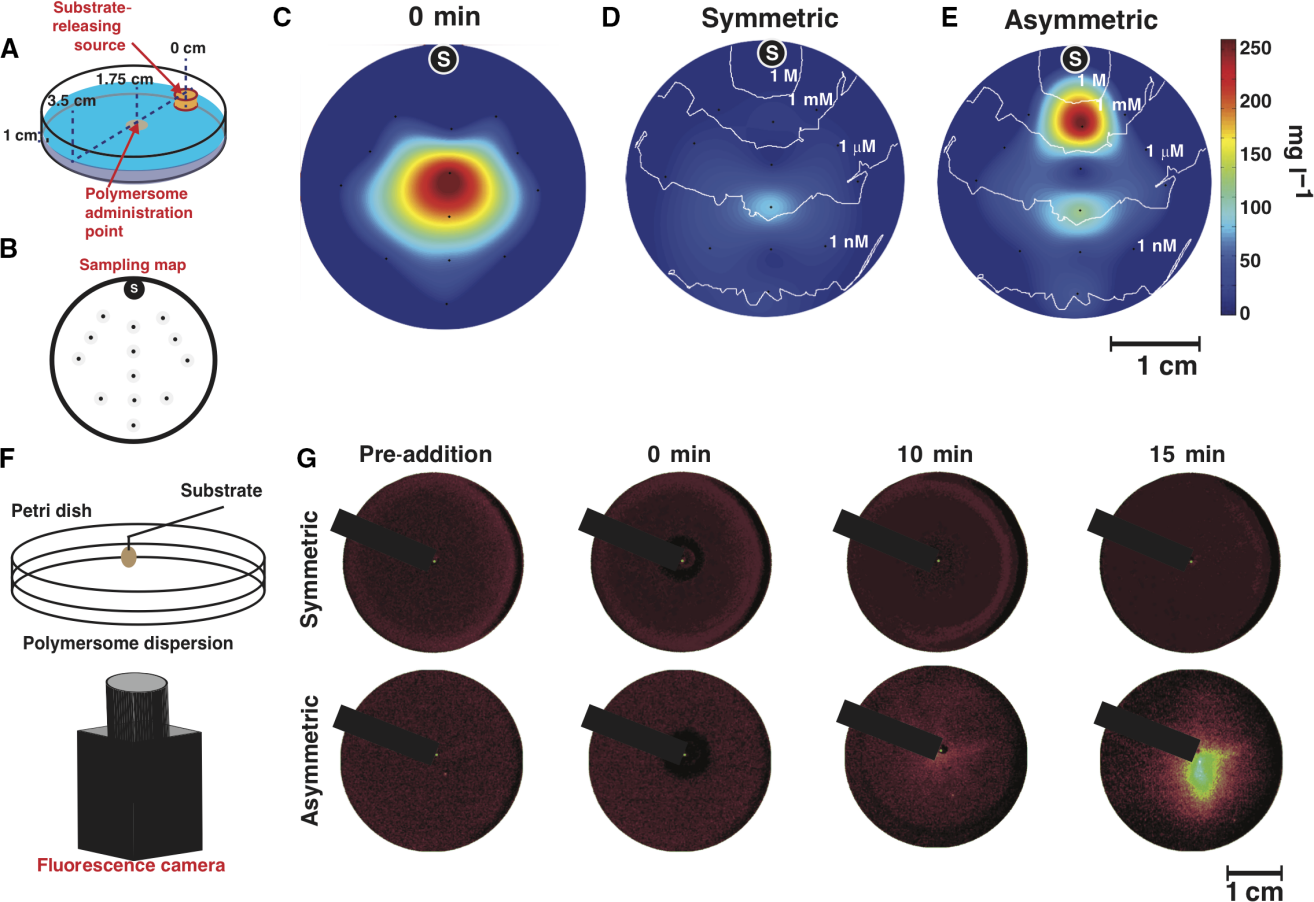
Collective chemotaxis. (**A**) Schematic of a petri dish where a cylindrical agarose gel soaked in glucose is placed. At time *t* = 0, a 1 mg ml^−1^ concentration of polymersomes is added in the dish center, and their concentration is sampled at different locations as indicated by the sampling map in (**B**). The dot labeled with “S” indicates the position of the source of glucose. (**C** to **E**) The resulting maps show the 2D distribution of asymmetric polymersomes (C) at time *t* = 0, and the distribution of polymersomes at time *t* = 10 min for (D) symmetrical PMPC-PDPA and (E) asymmetrical PMPC-PDPA/PEO-PBO polymersomes loaded with catalase and glucose oxidase. The isocratic white lines show the glucose gradient calculated by computational fluid dynamics. (**F**) A similar experiment is performed by adding glucose in the center of a petri dish containing fluorescently labeled polymersomes after they have thermalized in it. The imaging is performed with a fluorescence camera. (**G**) The corresponding fluorescence images are shown for both symmetric PMPC-PDPA and asymmetric PMPC-PDPA/PEO-PBO polymersomes loaded with catalase and glucose oxidase at different times: before the addition of glucose, at times *t* = 0, 10, and 15 min. The black bar indicates the needle for the injection of glucose over the imaging camera.

**Fig. 5:**
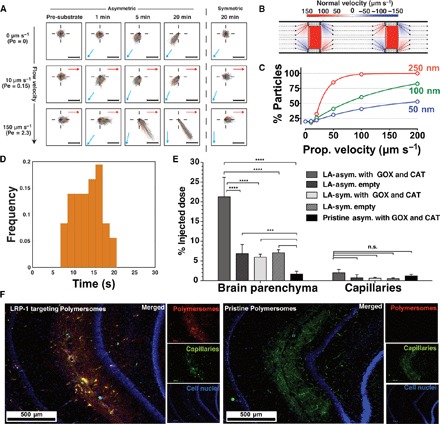
Chemotaxis under flow and in vivo. (**A**) Normalized polymersome 1-s trajectories measured in the presence of steady-state flow (0, 0.5, and 3.5 μm s^−1^) and collected before and 1, 5, and 20 min after the glucose gradient addition for both PMPC-PDPA/PEO-PBO asymmetric and PMPC-PDPA symmetric polymersomes loaded with glucose oxidase and catalase. Red arrows denote the direction of the flow within the observation area, whereas the blue arrows denote the average direction of the glucose gradient within it. Scale bars, 20 μm. Pe, Péclet number. (**B**) Streamlines of flow observed in a capillary with a radius of 4 μm and a length of 800 μm calculated by computational fluid dynamics. The red cylinders represent erythrocytes (hematocrit *H*% = 10.7%), and the color map shows the normal velocity of the flow, that is, the component perpendicular to the vessel walls. (**C**) Simulated percentage of the total number of particles bound to the vessel surface as a function of their drift velocity in a gradient for 50-, 100-, and 250-nm asymmetric nanoparticles calculated with an agent-based model of chemotactic particles within a capillary such as in (B). Note that the error bars show the SE. (**D**) Frequency distribution of the crossing time from apical to basolateral of LA–POEGMA-PDPA polymersomes measured over 35 different measurements using the in vitro BBB model as shown in fig. S18 (note that one example measurement is shown in fig. S19). (**E**) Percentage of the injected dose found in the rat brain parenchyma and the capillary fraction 10 min after carotid artery in situ perfusion of LA–POEGMA-PDPA/PBO asymmetric polymersomes loaded with Gox + Cat and empty and LA–POEGMA-PDPA symmetric polymersomes loaded with Gox + Cat and empty, as well as pristine asymmetric POEGMA-PDPA/PEO-PBO polymersomes loaded with Gox and Cat (*n* = 6; ***P < 0.001 and ****P < 0.0001). The error bars show the SE. n.s., not significant. (**F**) Immunofluorescence histologies of rat hippocampus sections of animals treated with LA–POEGMA-PDPA/PBO asymmetric polymersomes loaded with Gox + Cat and pristine asymmetric POEGMA-PDPA/PEO-PBO polymersomes loaded with Gox and Cat.

To get further insight into the chemotactic response of our system, we performed further experiments on the polymersomes loaded with both enzymes to more quantitatively assess their chemotactic capability using the approach shown in Fig. 4A. A cylindrical agarose gel presoaked in 1 M glucose solution was placed on the edge of a petri dish filled with PBS. Various polymersome formulations were added at the center of the dish with a syringe pump. Samples were collected at different locations within the petri dish and at different time points as shown in Fig. 4B and quantified for concentration and sizing (fig. S17 and note S3). In Fig. 4 (C to E), we show the concentration maps of the polymersomes in the dish at time 0 min (Fig. 4C) and 10 min after their addition, both for the symmetric formulation (Fig. 4D) and for the asymmetric formulation (Fig. 4E) loaded with glucose oxidase and catalase, in response to a glucose gradient. We also studied a different configuration (note S3): a petri dish prefilled with fluorescent polymersomes where a drop of 1 M glucose solution is added in the center of the dish, which is directly imaged with a fluorescence camera (Fig. 4F). The corresponding fluorescence images of both symmetric and asymmetric polymersomes before glucose addition and at times *t* = 0, 10, and 15 min are shown. Whereas the first experiment shows that the asymmetric polymersomes do not dilute in the presence of the glucose gradient and, instead, almost entirely drift toward the glucose source (Fig. 4E), in the second experiment, we can observe that the asymmetric polymersomes can concentrate toward the glucose gradient from high dilutions (Fig. 4G). These experiments show quite convincingly that the chemotactic polymersomes follow shallow gradients and concentrate toward a given chemical source over time scales of minutes and length scales 10^7^ times longer than the swimmer’s characteristic size. All the data bode well for bestowing polymersomes with chemotactic capability and augmenting their efficiency in navigating across biological barriers. To understand the effect of flow, we performed the same experiments as in Fig. 2 but in the presence of a constant flow almost perpendicular to the glucose gradient. The two chosen flow rates of 0.5 and 3.5 μl min^−1^, corresponding to velocities of 10 and 150 μm s^−1^ (that is, Péclet number of 0.15 and 2.3, respectively), represent conditions encountered next to the capillary barriers or right in the capillary center, respectively. As shown in Fig. 5A, the normalized trajectories for both pre–substrate addition and symmetric polymersomes show a typical Gaussian distribution that is more skewed as the flow rate increases from 0 to 3.5 μl min^−1^. At zero flow, the glucose oxidase– and catalase-loaded polymersomes show a rapid response to the glucose gradient with overall drift plateauing at about 20 min after the addition of glucose. At a flow rate of 0.5 μl min^−1^, the chemotactic drift is still sufficiently large to overcome the convection, and indeed, polymersomes still move toward the glucose gradient, albeit at lower velocities. At a flow rate of 3.5 μl min^−1^, the chemotactic drift combines with the flow, inducing a drift of the polymersomes with trajectories taking a direction of about 45° from the flow line. It is important to note (as shown in Fig. 5A) that as the flow increases, the gradient vector rotates from its original unbiased position to being almost perpendicular to the flow. To test the effect of placing chemotactic polymersomes in blood flow, we used an agent-based model of the nanoparticles in capillaries in the presence of erythrocytes (also known as red blood cells) that we have developed previously (*48*). In Fig. 5B, we show a snapshot of the streamlines of the flow observed in a capillary with a radius of 4 μm and a length of 800 μm, calculated by computational fluid dynamics (note S3). The red cylinders represent erythrocytes (at physiological hematocrit *H*% = 10.7%), and the color maps show the normal velocity, that is, the velocity component perpendicular to the vessel wall. We used this geometry and seeded 100 nanoparticles randomly at the entrance of the vessel and allowed their passage through the vessel. The vessel walls were set as no-slip, sticky boundaries (that is, as a polymersome approaches the barrier it binds to it), so that the number of nanoparticles bound to the vessel wall could be evaluated with different-sized particles and velocities of propulsion. As discussed above, we can assume that as asymmetric polymersomes encounter a glucose gradient, they will propel with a propulsion velocity that is directly proportional to the gradient, and their rotation is uniquely controlled by Brownian dynamics. Assuming a glucose gradient across the vessel, we performed the calculations for polymersomes with radius *R* = 50, 100, and 250 nm, which is representative of a typical size distribution of polymersomes (see DLS-measured distributions in fig. S1), and to represent the spread of propulsion velocities (see both Figs. 2 and 3), we propelled the polymersomes from 0 to 200 μm s^−1^. Figure 5C shows the percentage of particles that bind to the vessel wall during a single passage. Binding to the vessel walls is generally improved by increasing the propulsion velocity. Propulsion augments binding twofold from 0 to 200 μm s^−1^ for small nanoparticles, and the binding to the wall is considerably improved for the case of larger polymersomes and high propulsion velocity reaching almost 100% of particles binding. Bigger particles bind better to the wall than smaller particles do due to their smaller rotational diffusion, which keeps the particles’ orientation along the gradient for longer (*12*). Modeling would thus suggest that adding an element of propulsion to the motion of the polymersomes increases the overall uptake from the blood due to their improved distribution to the endothelial wall interface. Furthermore, the use of glucose as a substrate ensures that there is a high level of substrate available within the blood, because blood glucose is maintained at 4 to 7.8 mM (*24*). In addition, brain metabolism requires high levels of glucose, and glucose transporters are well known to be overexpressed on the BBB (*24*), and hence, it is not farfetched to assume that blood glucose has a positive gradient toward the blood wall and an even more favorable distribution within the brain. Recently, we have demonstrated that polymersomes can be conjugated with peptides that target the LRP-1 receptor. This receptor is overexpressed at the BBB, and it is associated with a transport mechanism known as transcytosis. We have demonstrated that, by targeting this pathway, we can deliver large macromolecules to CNS resident cells (*27*). LA-modified asymmetric polymersomes can cross the BBB, and we showed this using a 3D in vitro BBB model that comprises two cell types: brain endothelial cells and pericytes cultured in the presence of conditioned medium from astrocytes. The endothelial cells are placed on the upper compartment, and they are separated from the pericytes by a porous polycarbonate membrane (pores < 0.4 μm) (*27*). The geometry of the model is shown in fig. S18A alongside with the qualitative (fig. S18B) and quantitative (fig. S18C) kinetics of the polymersome BBB crossing. These data show effective crossing and active pumping of the LA polymersomes from the apical to the basolateral side of the BBB performed by the endothelial cells. Moreover, the same in vitro model can be used to evaluate the early time points, and as shown in Fig. 5D and fig. S19, we observed that LRP-1–mediated transcytosis is extremely fast, taking about 15 s from the binding event on the apical side to a full crossing to the basolateral side. We have used this system to demonstrate that chemotaxis can augment delivery significantly. This effect was validated in the rat CNS through in situ brain perfusion and quantification of fluorescently labeled polymersomes in the different parts of the brain by fractionation. Chemotactic polymersomes, responsive to glucose and functionalized with LA, demonstrated about a fourfold delivery increase into the parenchyma compared to nonchemotactic polymersome controls, including LA-modified asymmetric empty polymersomes and LA-symmetric polymersomes either loaded with Gox + Cat or empty (Fig. 5E). The effective passage across the BBB is further demonstrated by immunofluorescence histologies of the brain sections whose capillaries are stained using the CD34 marker (green); the cell nuclei are stained with Hoechst (blue), and the polymersomes are labeled with Cy3 (red) as shown in Fig. 5F. The nonactive polymersomes were optimized to reach a respectable 5% of the injected dose. However, modifying the polymersomes, by adding an asymmetric patch and loading them with glucose oxidase and catalase, enabled a staggering delivery of 20% of the injected dose, which, to the best of our knowledge, has never been reported so far with any other system. The glucose is a required metabolite in the blood, and the brain consumes more than 20% of the assimilated glucose at any given time. It is also established that the brain endothelial cells express extremely high level of glucose transporters (*49*), suggesting that as the blood reach the brain area, there must be a gradient from the center to the wall of the vessel.

## Conclusions

We have shown here that an established intracellular delivery system such as PMPC-PDPA and POEGMA-PDPA polymersomes can be modified to have chemotactic capabilities toward glucose gradients. We achieve this by using a novel process of converting a chemical potential difference into an actual propulsion mechanism capable of tracking small-molecule gradients over distances that are many orders of magnitude greater than the nanoparticle’s characteristic length. We demonstrated that nanoscopic polymersomes move according to superdiffusional behaviors, and they do so only in the presence of a gradient becoming chemotactic. This is achieved by protecting the actual molecular machinery (the enzymes) within the polymersome aqueous lumen away from immunological signaling and proteolytic degradations. We show that such a physical encapsulation enables high flexibility, and we show that self-phoresis can be achieved using different combinations of enzymes and substrates, with the only limiting factor being the ability of the substrate to penetrate across the polymersome membrane. We have shown that the combination of glucose oxidase and catalase makes a very efficient chemotactic polymersome in the presence of a glucose gradient. Glucose oxidase and catalase work in tandem to create propulsion, transforming endogenous occurring glucose to endogenous occurring d-glucono-δ-lactone and water, without the formation of potentially harmful compounds such as hydrogen peroxide and gaseous oxygen. Finally, we demonstrate that with very minimal modification, we transform a well-established delivery system, the polymersome, into an efficient carrier that enables for the first time the use of chemotaxis to augment biological barrier crossing. This is proved by augmenting the delivery across the BBB, where we have demonstrated an increase of almost fourfold in the amount of polymersomes gaining access to the brain parenchyma of rats compared to BBB-targeting, nonchemotactic polymersomes. This is a strong finding that we envision will set a completely new trend in the design of drug delivery systems embracing the new advances being proposed in active colloids.

## MATERIALS AND METHODS

### Materials

Chemicals were used as received unless otherwise indicated. 2-(Methacryloyloxy)ethyl phosphorylcholine (MPC > 99%) was donated by Biocompatibles. 2-(Diisopropylamino)ethyl methacrylate (DPA) was purchased from Scientific Polymer Products. Copper(I) bromide (CuBr; 99.999%), 2,2′-bipyridine (bpy), methanol (anhydrous, 99.8%), and isopropanol were purchased from Sigma-Aldrich. The silica used for the removal of the atom transfer radical polymerization (ATRP) copper catalyst was column chromatography grade silica gel 60 (0.063 to 0.200 mm) purchased from E. Merck. 2-(*N*-morpholino)ethyl 2-bromo-2-methylpropanoate (ME-Br) initiator was synthesized according to a previously reported procedure (*50*). Poly(ethylene glycol) methyl ether methacrylate [P(OEG_10_MA)] was purchased from Sigma-Aldrich. PEO-PBO copolymer was purchased from Advanced Polymer Materials Inc. The polymersomes were labeled using rhodamine B octadecyl ester perchlorate purchased from Sigma-Aldrich. PBS was made from Oxoid tablets (one tablet per 100 ml of water). Bovine liver catalase, glucose oxidase, and glucose have been purchased from Sigma-Aldrich. The gel filtration column for the purification of the polymersomes was made with Sepharose 4B purchased from Sigma-Aldrich.

### PMPC_25_-PDPA_70_ copolymer synthesis

The PMPC-*b*-PDPA diblock copolymer was prepared by ATRP (*50*). In a typical ATRP procedure, a Schlenk flask with a magnetic stir bar and a rubber septum was charged with MPC (1.32 g, 4.46 mmol) and ME-Br initiator (50.0 mg, 0.178 mmol) in ethanol (4 ml) and purged for 30 min with N_2_. CuBr (25.6 mg, 0.178 mmol) and bpy ligand (55.8 mg, 0.358 mmol) were added as a solid mixture into the reaction flask. The [MPC]/[ME-Br]/[CuBr]/[bpy] relative molar ratios were 25:1:1:2. The reaction was carried out under a nitrogen atmosphere at 20°C. After 60 min, deoxygenated DPA (6.09 g, 28.6 mmol) and methanol (7 ml) mixture was injected into the flask. After 48 hours, the reaction solution was diluted by the addition of ethanol (about 200 ml) and then passed through a silica column to remove the copper catalyst. The reaction mixture was dialyzed against water to remove the organic solvent and then freeze-dried. Finally, the copolymer molecular weight was checked by nuclear magnetic resonance (NMR) analysis.

### P(OEG_10_MA)_20_-PDPA_100_ copolymer synthesis

The protected maleimide initiator (Mal-Br) was prepared according to a previously published procedure (*51*). In a typical procedure, either ME-Br or Mal-Br initiators or ATRP initiators (0.105 mmol, 1 equiv.) were mixed with OEG_10_MA (1 g, 2.11 mmol, 20 equiv.). When homogeneous, 1 ml of water was added, and the solution was purged with nitrogen for 40 min. Then, a mixture of CuCl (10.4 mg, 0.105 mmol) and bpy (32.9 mg, 0.210 mmol) was mixed. After 8 min, a sample was removed, and a nitrogen-purged mixture of DPA (2.2455 g, 0.0105 mol, 100 equiv.) mixed with 3 ml of isopropanol was added to the viscous mixture via cannula. After 18 hours, the mixture was diluted with methanol. Then, two volumes of dichloromethane were added. The solution was passed through a column of silica using dichloromethane/methanol (2:1) to remove the copper catalyst. The resulting solution was dialyzed [molecular weight cutoff (MWCO) 1000] against ethanol and water and freeze-dried. The resulting copolymer composition was determined by NMR analysis.

### Copolymer conjugation with cysteine-terminated peptide

We dispersed the deprotected maleimide P(OEG_10_MA)_20_-PDPA_100_ (105.6 mg, ≅3.4 μmol of maleimide) in 4.5 ml of nitrogen-purged PBS at pH 7.3. The pH was lowered by the addition of concentrated HCl (10 μl) to give a uniform solution. The pH was then increased to 7.8 with 5 M NaOH, and the resulting opaque dispersion was sonicated for 10 min. This solution (2.3 ml) was transferred to a second flask. Both solutions were then purged with nitrogen for 10 min (this should give an approximate maleimide amount in each flask of 1.7 μmol). To the original solution, we added Cys-Angiopep (5.5 mg, 2.3 μmol of thiol) followed by tris(2-carboxyethyl)phosphine (2 mg, 7 μmol). The pH in each solution was measured to 7. Both solutions were left for 17 hours. Then, both solutions were dialyzed against water (MWCO 8000) to remove any excess peptide, followed by freeze-drying. Successful labeling was confirmed using a high-performance liquid chromatography (HPLC) with fluorescence and absorption detection (contains fluorescent tyrosine residues), rendering the polymer-peptide conjugates fluorescent at 303 nm when excited at 274 nm. On the other hand, the nonlabeled polymer does not exhibit any fluorescence at these wavelengths (but can be detected using the absorption detector).

### Polymersome preparation

Nanometer-sized polymersomes were formed by the film rehydration method (*52*, *53*). The block copolymers were dissolved in 2:1 (v/v) chloroform/methanol at a total copolymer concentration of 10 mg ml^−1^ in the organic solvent. Asymmetric polymersomes were obtained by dissolving premixed copolymers at 90% PMPC_25_-PDPA_70_ or P(OEG_10_MA)_20_-PDPA_100_ and 10% PEO_16_-PBO_22_ in molar ratio. Rhodamine B in chloroform solution was added to the above solutions to create a fluorophore final concentration of 50 μg ml^−1^. Polymeric films were obtained by drying the copolymer solutions in vacuum oven overnight. In a typical experiment, 0.1 M PBS (pH 7.4) was added to the polymeric films, and they were let stir for 30 days at room temperature to obtain the formation of PEO-PBO domains on the PMPC-PDPA polymersomes surface. Topological asymmetry and size distribution have been characterized by TEM and DLS analysis, respectively.

### Transmission electron microscopy

A PTA solution was used as a positive and a negative staining agent because of its preferential interaction with the ester groups on the PMPC polymers (*54*), which are not present in the PEO-PBO copolymer. The PTA staining solution was prepared by dissolving 37.5 mg of PTA in boiling distilled water (5 ml). The pH was adjusted to 7.4 by adding a few drops of 5 M NaOH with continuous stirring. The PTA solution was then filtered through a 0.2-μm filter. Then, 5 μl of polymersome/PBS dispersion was deposited onto glow-discharged copper grids. After 1 min, the grids were blotted with filter paper and then immersed into the PTA staining solution for 5 s for positive staining and 10 s for negative staining. Then, the grids were blotted again and dried under vacuum for 1 min. Grids were imaged using an FEI Tecnai G2 Spirit TEM microscope at 80 kV.

### Dynamic light scattering

The sample was crossed by a 120-mW He-Ne laser at 630 nm at a controlled temperature of 25°C, and the scattered light was measured at an angle of 173°. For the analysis, the sample was diluted with filtered PBS (pH 7) at a final concentration of 0.2 mg ml^−1^ into a final volume of 500 μl and then analyzed into a polystyrene cuvette (Malvern, DTS0012). All DLS data were processed using a Dispersion Technology Software (Malvern Instruments).

### Reversed-phase HPLC

Reversed-phase HPLC (RP-HPLC) was performed with Dionex UltiMate 3000 instrument equipped with variable wavelength detector to analyze the ultraviolet absorption of the polymers at 220 nm and the enzyme signal at 280 nm. A gradient of H_2_O + trifluoroacetic acid was 0.05.

### Enzyme encapsulation

Electroporation was used to allow the entrapment of glucose oxidase, catalase, or the combination of the two within the polymersomes. The optimal setting used for the electroporation was 10 pulses at 2500 V (*30*). The number of enzymes that can be encapsulated is dictated by the enzyme charge and size. As we demonstrated previously (*30*), the loading can be modulated by changing the electroporation ac voltage intensity and the number of pulses and by adjusting the enzyme surface charges (for example, controlling the solution pH). After electroporation, the samples were purified by preparative gel permeation chromatography. Then, the amount of polymer and encapsulated enzymes was quantified by RP-HPLC.

### Encapsulation efficiency calculation

HPLC and DLS data were combined to calculate the number of polymersomes produced in any experiment. The encapsulation efficiency was defined as the number of molecules of enzyme loaded in each polymersomes. The number of polymersomes in a sample can be estimated from the aggregation number (*N*_agg_), which is defined as

$$N_{\text{agg}}=\frac{4}{3}\pi \frac{{\left(R-l_{b}\right)}^{3}-{\left(R-l_{b}-t_{m}\right)}^{3}}{\nu_{\text{PDPA}}}$$ (3)

where *R* is the particle radius from the DLS, *l*_b_ is the length of the hydrophilic PMPC brush, *t*_m_ is the thickness of the PDPA membrane, and ν_PDPA_ is the molecular volume of a single PDPA chain. The number of polymersomes (*N*_ps_) in the sample is defined as

$$N_{\text{ps}}=\sum_{i=0}^{n}N_{\text{agg}}[P]N_{a}\Phi_{i}R_{i}$$ (4)

where [*P*] is the moles of copolymer in the sample, *N*_a_ is Avogadro’s number, and Φ_*i*_*R*_*i*_ is the fraction of sample at a defined radius *R*. Finally, the encapsulation efficiency *e* is given by

$$e=\frac{N_{e}}{N_{\text{ps}}}$$ (5)

where *N*_e_ is the number of enzymes in the sample. The average of encapsulated enzymes per polymersome was 1.9 ± 0.25 for the catalase and 6 ± 0.45 for the glucose oxidase. Results are shown in fig. S1 and table S1.

### NTA measurements of polymersomes diffusion

NTA was performed with a NanoSight LM14 instrument equipped with a Scientific CMOS camera mounted on an optical microscope to track scattered light by particles illuminated by a focused (80 μm) beam generated by a single mode laser diode (405 nm). The polymersome solution (1 ml) was injected in a concentration of approximately 100 particles/ml in PBS. Samples and controls were injected into the NanoSight chamber as described in fig. S2. Two different populations of polymersomes (asymmetric and symmetric) were analyzed with hydrogen peroxide/glucose, depending on the loaded enzyme. Particles were tracked by the built-in software for 60 seconds at 30 fps. The recorded tracks were analyze using Matlab. Origin of movement for all particles was normalized to Cartesian coordinates (0,0). The MSD of all particles was calculated as reported in Volpe *et al*. (*43*). Tracks were analyzed for 1 s. Particles not tracked for at least 1 s were discarded from the analysis. The average number of tracks per sample ranged from 2000 to 10,000 traces.

### In vitro 3D cell culture BBB

For mouse brain endothelial cells [bEnd.3, American Type Culture Collection (ATCC) CRL-2299], the medium used was Dulbecco’s modified Eagle’s medium (DMEM) supplemented with 10% fetal calf serum (FCS), penicillin and streptomycin, l-glutamine, and fungizone. Astrocyte (ATCC CRL-2541, C8-D1A Astrocyte type I clone) medium was antibiotic-free DMEM supplemented with 10% FCS and l-glutamine. Pericyte (mesenchymal stem cell, Gibco iMouse, C57BL/6) medium used was DMEM F12 medium with GlutaMAX-I, supplemented with 10% FCS and gentamicin (5 μg ml^−1^). For transwell experiments, both sides of the Transwell insert filters (Corning 3460 polyethylene filter; diameter, 1.05 cm; pore size, 0.4 μm) were precoated with collagen (10 μg cm^−2^) for 2 hours at room temperature. This was followed by seeding bEnd.3 endothelial cells on the upper surface of the Transwell at a density of 20,000 to 40,000 cells per well and incubated for 12 hours at 37°C in 95% air and 5% CO_2_ to allow the cells to fully attach. Next, pericytes (10,000 to 20,000 cells per well) were seeded on the opposite side of the filter insert and incubated for 12 hours at 37°C in 95% air and 5% CO_2_. Finally, the inserts were moved to a Transwell plate and incubated for 7 days at 37°C, with the medium being changed every 2 days. Note that the medium was supplemented with conditioned medium extracted from the astrocyte culture. The endothelial tight junctions were stained with either anti–ZO-1 or claudin-5, whereas pericytes were shown using anti-CD140. For confocal imaging, the BBB models were fixed and imaged using a *z* stack of 100 images with an optical slice of 0.4 μm. The concentration of polymersomes on the upper (apical) and lower (basolateral) compartments was measured by HPLC using fluorescence detectors collecting samples at different time points. For the early time point and live cell kinetics, brain endothelial cells were treated with CellMASK for 30 min and washed three times with PBS and immersed in imaging medium (FluoroBrite DMEM) supplemented with 10% FCS and gentamicin (5 μg ml^−1^). Polymersomes were subsequently added at a concentration of 1 mg ml^−1^ into the apical (upper) Transwell compartment after transepithelial electric resistance measurements were taken with an EVOM2 epithelial voltohmmeter. Cells were incubated for 1 to 2 hours at 37°C in 95% air and 5% CO_2_ and imaged on Leica SP8 confocal laser scanning microscope with 40× water immersion lens and 63× oil immersion lens. Rhodamine-labeled polymersomes with an excitation wavelength of 561 nm was used, and fluorescence emission was measured at a wavelength of 575 to 600 nm. Cells’ membrane was stained with CellMASK. Image data were acquired and processed using ImageJ software. We repeated this experiment three times and measured a total of 35 crossing events

### Brain in situ perfusion

Male adult Wistar rats were anaesthetized with ketamine (100 mg kg^−1^) and medetomidine (1 mg kg^−1^) via intraperitoneal injection. The right and left external carotid arteries were isolated from the carotid sheaths and cannulated according to a previously established procedure (*55*). The perfusion fluid was a modified Ringer’s solution [NaCl (6.896 g liter^−1^), KCl (0.350 g liter^−1^), CaCl_2_ (0.368 g liter^−1^), MgSO_4_ (0.296 g liter^−1^), NaHCO_3_ (2.1 g liter^−1^), KH_2_O_4_ (0.163 g liter^−1^), and Hepes (2.383 g liter^−1^), with glucose (0.5005 g liter^−1^, 5.5 mM) and bovine serum albumin (BSA) (11.1 g liter^−1^)]. The perfusion fluid was bubbled with 5% CO_2_ and heated to 37°C for 20 min before perfusion. For the injection of polymersomes, 20% (mol) Cy3-labeled polymersomes in PBS with or without protein encapsulation were diluted to 1 mg ml^−1^ in Krebs buffer (pH 7.4) [188 mM NaCl, 4.7 mM KCl, 2.5 mM CaCl_2_, 1.2 mM MgSO_4_, 1.2 mM KH_2_PO_4_, 25 mM NaHCO_3_, 10 mM d-glucose, and BSA (3 g liter^−1^)]. The polymersome solution was supplied via syringe pump at 0.16 ml min^−1^, with a total perfusion rate of 1.5 ml min^−1^ and a total perfusion time of 10 min. At the end of the perfusion time, the syringe pump was stopped, and the arteries were flushed for 60 s with a modified Ringer’s perfusate to remove unbound polymersomes. After 60 s, cerebrospinal fluid was extracted via cisternal puncture followed by decapitation and removal of the brain.

### Quantification of polymersome distribution in the rat brain

After decapitation, brains were removed and washed in ice-cold NaCl (9 g liter^−1^), followed immediately by homogenization on ice to initiate the capillary depletion method (*55*). Briefly, the cerebellum was removed, and the cerebrum was weighed, adding 2× brain weight in PBS followed by 3× dilution in 30% (w/v) dextran (average MW 64,000 to 74,000). Centrifugation of homogenates at 7400*g* for 20 min in 4°C resulted in several fractions that were carefully separated: capillary-depleted (CD) fraction (that is, parenchyma), dextran, and the capillary-enriched fraction (pellet). The capillary-enriched pellet was resuspended in PBS, and 100 μl of the samples was added to a black 96-well plate and read in a fluorimeter at an excitation wavelength of 540 nm and emission at 565 nm. All sample fluorescence readings were normalized to readings obtained from sham perfused rats (*n* = 6) for each sample type, that is, CD, dextran, or capillaries. Positive controls were polymersomes in perfusate harvested from the cannula at the injection point. Normalized fluorescence readings were converted to a polymersome (Cy3) amount that was converted into percentage injected dose %*D* of the positive control value for that experiment, where %*D* = *d*_s_/*d*_p_%, where *d*_s_ is the normalized sample value (mg) and *d*_p_ is the mean positive control value (mg). This was further converted into fluorescence per whole brain. All statistical analyses were one-way analysis of variance (ANOVA), *P* < 0.05. All animal studies were carried out according to the ARRIVE (Animal Research: Reporting of In Vivo Experiments) guidelines under licence from the UK Home Office (Scientific Procedures Act 1986) and approved by the King’s College London ethical review committee.

## SUPPLEMENTARY MATERIALS

Supplementary material for this article is available at http://advances.sciencemag.org/cgi/content/full/3/8/e1700362/DC1 fig. S1. Number of encapsulated enzyme and polymersome size distribution. fig. S2. Schematic representation of the observation NanoSight chamber. fig. S3. Asymmetric polymersomes loaded with catalase in the presence of homogeneously dissolved hydrogen peroxide. fig. S4. Asymmetric polymersomes loaded with glucose oxidase in the presence of homogeneously dissolved glucose. fig. S5. Asymmetric polymersomes loaded with catalase and glucose oxidase in the presence of homogeneously dissolved glucose. fig. S6. Symmetric polymersomes loaded with catalase and glucose oxidase after the injection of PBS (that is, no gradient). fig. S7. Asymmetric empty polymersomes after the injection of PBS (that is, no gradient). fig. S8. Asymmetric polymersomes loaded with catalase and glucose oxidase after the injection of PBS (that is, no gradient). fig. S9. Symmetric PMPC-PDPA polymersomes loaded with catalase and glucose oxidase in the presence of a glucose gradient. fig. S10. Symmetric PEO-PBO polymersomes loaded with catalase and glucose oxidase in the presence of a glucose gradient. fig. S11. Asymmetric empty polymersomes in the presence of a glucose gradient. fig. S12. Asymmetric empty polymersomes in the presence of a hydrogen peroxide gradient. fig. S13. Asymmetric polymersomes loaded with catalase in the presence of a hydrogen peroxide gradient. fig. S14. Asymmetric polymersomes loaded with glucose oxidase in the presence of a glucose gradient. fig. S15. Asymmetric polymersomes loaded with catalase and glucose oxidase in the presence of a glucose gradient. fig. S16. Asymmetric PEO-PBO polymersomes loaded with catalase and glucose oxidase in the presence of a glucose gradient. fig. S17. Polymersome concentration in long-range chemotaxis. fig. S18. BBB in vitro model for polymersome qualification. fig. S19. Live cell imaging of LA polymersome real-time transcytosis. table S1. Diffusion simulation parameters. note S1. An agent-based model of nanoparticle propulsion in a capillary. note S2. Long-range chemotaxis. note S3. Simulation model and propulsion velocity fitting. note S4. Diffusion simulations.

## References

[1] Vorotnikov A. V., Chemotaxis: Movement, direction, control. Biochemistry 76, 1528–1555 (2011). 22339602 10.1134/S0006297911130104

[2] Parent C. A., Devreotes P. N., A cell’s sense of direction. Science 284, 765–770 (1999). 10221901 10.1126/science.284.5415.765

[3] Porter S. L., Wadhams G. H., Armitage J. P., Signal processing in complex chemotaxis pathways. Nat. Rev. Microbiol. 9, 153–165 (2011). 21283116 10.1038/nrmicro2505

[4] Son K., Brumley D. R., Stocker R., Live from under the lens: Exploring microbial motility with dynamic imaging and microfluidics. Nat. Rev. Microbiol. 13, 761–775 (2015). 26568072 10.1038/nrmicro3567

[5] Zou Y.-R., Kottmann A. H., Kuroda M., Taniuchi I., Littman D. R., Function of the chemokine receptor CXCR4 in haematopoiesis and in cerebellar development. Nature 393, 595–599 (1998). 9634238 10.1038/31269

[6] Junger W. G., Immune cell regulation by autocrine purinergic signalling. Nat. Rev. Immunol. 11, 201–212 (2011). 21331080 10.1038/nri2938 PMC4209705

[7] Roussos E. T., Condeelis J. S., Patsialou A., Chemotaxis in cancer. Nat. Rev. Cancer 11, 573–587 (2011). 21779009 10.1038/nrc3078 PMC4030706

[8] Dusenbery D. B., Minimum size limit for useful locomotion by free-swimming microbes. Proc. Natl. Acad. Sci. U.S.A. 94, 10949–10954 (1997). 9380740 10.1073/pnas.94.20.10949 PMC23542

[9] Ebbens S. J., Howse J. R., In pursuit of propulsion at the nanoscale. Soft Matter 6, 726–738 (2010).

[10] Sanchez S., Soler L., Katuri J., Chemically powered micro- and nanomotors. Angew. Chem. Int. Ed. 54, 1414–1444 (2015). 10.1002/anie.201406096 25504117

[11] Yadav V., Duan W., Butler P. J., Sen A., Anatomy of nanoscale propulsion. Annu. Rev. Biophys. 44, 77–100 (2015). 26098511 10.1146/annurev-biophys-060414-034216

[12] Bechinger C., Di Leonardo R., Löwen H., Reichhardt C., Volpe G., Volpe G., Active particles in complex and crowded environments. Rev. Mod. Phys. 88, 045006 (2016).

[13] Purcell E. M., Life at low Reynolds number. Am. J. Phys. 45, 3–11 (1977).

[14] Dreyfus R., Baudry J., Roper M. L., Fermigier M., Stone H. A., Bibette J., Microscopic artificial swimmers. Nature 437, 862–865 (2005). 16208366 10.1038/nature04090

[15] Anderson J. L., Colloid transport by interfacial forces. Annu. Rev. Fluid Mech. 21, 61–99 (1989).

[16] Derjaguin B. V., Sidorenkov G. P., Zubashchenkov E. A., Kiseleva E. V., Kinetic phenomena in boundary films of liquids. Kolloidn. Zh. 9, 335–347 (1947).

[17] Golestanian R., Liverpool T. B., Ajdari A., Propulsion of a molecular machine by asymmetric distribution of reaction products. Phys. Rev. Lett. 94, 220801 (2005). 16090376 10.1103/PhysRevLett.94.220801

[18] Howse J. R., Jones R. A. L., Ryan A. J., Gough T., Vafabakhsh R., Golestanian R., Self-motile colloidal particles: From directed propulsion to random walk. Phys. Rev. Lett. 99, 048102 (2007). 17678409 10.1103/PhysRevLett.99.048102

[19] Hong Y., Blackman N. M. K., Kopp N. D., Sen A., Velegol D., Chemotaxis of nonbiological colloidal rods. Phys. Rev. Lett. 99, 178103 (2007). 17995374 10.1103/PhysRevLett.99.178103

[20] Gao W., Dong R., Thamphiwatana S., Li J., Gao W., Zhang L., Wang J., Artificial micromotors in the mouse’s stomach: A step toward in vivo use of synthetic motors. ACS Nano 9, 117–123 (2015). 25549040 10.1021/nn507097k PMC4310033

[21] Abbott N. J., Patabendige A. A. K., Dolman D. E. M., Yusof S. R., Begley D. J., Structure and function of the blood–brain barrier. Neurobiol. Dis. 37, 13–25 (2010). 19664713 10.1016/j.nbd.2009.07.030

[22] Abbott N. J., Rönnbäck L., Hansson E., Astrocyte–endothelial interactions at the blood–brain barrier. Nat. Rev. Neurosci. 7, 41–53 (2006). 16371949 10.1038/nrn1824

[23] Navarrete A., van Schaik C. P., Isler K., Energetics and the evolution of human brain size. Nature 480, 91–93 (2011). 22080949 10.1038/nature10629

[24] Mergenthaler P., Lindauer U., Dienel G. A., Meisel A., Sugar for the brain: The role of glucose in physiological and pathological brain function. Trends Neurosci. 36, 587–597 (2013). 23968694 10.1016/j.tins.2013.07.001 PMC3900881

[25] Lomas H., Canton I., MacNeil S., Du J., Armes S. P., Ryan A. J., Lewis A. L., Battaglia G., Biomimetic pH sensitive polymersomes for efficient DNA encapsulation and delivery. Adv. Mater. 19, 4238–4243 (2007).

[26] Messager L., Gaitzsch J., Chierico L., Battaglia G., Novel aspects of encapsulation and delivery using polymersomes. Curr. Opin. Pharmacol. 18, 104–111 (2014). 25306248 10.1016/j.coph.2014.09.017

[27] Tian X., Nyberg S., Sharp P. S., Madsen J., Daneshpour N., Armes S. P., Berwick J., Azzouz M., Shaw P., Abbott N. J., Battaglia G., Lrp-1-mediated intracellular antibody delivery to the central nervous system. Sci. Rep. 5, 11990 (2015). 26189707 10.1038/srep11990 PMC4507173

[28] Discher B. M., Won Y.-Y., Ege D. S., Lee J. C.-M., Bates F. S., Discher D. E., Hammer D. A., Polymersomes: Tough vesicles made from diblock copolymers. Science 284, 1143–1146 (1999). 10325219 10.1126/science.284.5417.1143

[29] Gaitzsch J., Appelhans D., Wang L., Battaglia G., Voit B., Synthetic bio-nanoreactor: Mechanical and chemical control of polymersome membrane permeability. Angew. Chem. Int. Ed. 51, 4448–4451 (2012). 10.1002/anie.201108814 22438056

[30] Wang L., Chierico L., Little D., Patikarnmonthon N., Yang Z., Azzouz M., Madsen J., Armes S. P., Battaglia G., Encapsulation of biomacromolecules within polymersomes by electroporation. Angew. Chem. Int. Ed. 51, 11122–11125 (2012). 10.1002/anie.201204169 23023772

[31] Massignani M., LoPresti C., Blanazs A., Madsen J., Armes S. P., Lewis A. L., Battaglia G., Controlling cellular uptake by surface chemistry, size, and surface topology at the nanoscale. Small 5, 2424–2432 (2009). 19634187 10.1002/smll.200900578

[32] LoPresti C., Massignani M., Fernyhough C., Blanazs A., Ryan A. J., Madsen J., Warren N. J., Armes S. P., Lewis A. L., Chirasatitsin S., Battaglia G., Controlling polymersome surface topology at the nanoscale by membrane confined polymer/polymer phase separation. ACS Nano 5, 1775–1784 (2011). 21344879 10.1021/nn102455z

[33] Battaglia G., LoPresti C., Massignani M., Warren N. J., Madsen J., Forster S., Vasilev C., Hobbs J. K., Armes S. P., Chirasatitsin S., Engler A. J., Wet nanoscale imaging and testing of polymersomes. Small 7, 2010–2015 (2011). 21695783 10.1002/smll.201100511 PMC3325755

[34] Ruiz-Perez L., Messager L., Gaitzsch J., Joseph A., Sutto L., Gervasio F. L., Battaglia G., Molecular engineering of polymersome surface topology. Sci. Adv. 2, e1500948 (2016). 27152331 10.1126/sciadv.1500948 PMC4846435

[35] Ruiz-Pérez L., Madsen J., Themistou E., Gaitzsch J., Messager L., Armes S. P., Battaglia G., Nanoscale detection of metal-labeled copolymers in patchy polymersomes. Polym. Chem. 6, 2065–2068 (2015).

[36] Simón-Gracia L., Hunt H., Scodeller P. D., Gaitzsch J., Braun G. B., Willmore A.-M. A., Ruoslahti E., Battaglia G., Teesalu T., Paclitaxel-loaded polymersomes for enhanced intraperitoneal chemotherapy. Mol. Cancer Ther. 15, 670–679 (2016). 26880267 10.1158/1535-7163.MCT-15-0713-T PMC4873343

[37] Murdoch C., Reeves K. J., Hearnden V., Colley H., Massignani M., Canton I., Madsen J., Blanazs A., Armes S. P., Lewis A. L., Battaglia G., Internalization and biodistribution of polymersomes into oral squamous cell carcinoma cells in vitro and in vivo. Nanomedicine 5, 1025–1036 (2010). 20874018 10.2217/nnm.10.97

[38] Colley H. E., Hearnden V., Avila-Olias M., Cecchin D., Canton I., Madsen J., MacNeil S., Warren N., Hu K., McKeating J. A., Armes S. P., Murdoch C., Thornhill M. H., Battaglia G., Polymersome-mediated delivery of combination anticancer therapy to head and neck cancer cells: 2D and 3D in vitro evaluation. Mol. Pharm. 11, 1176–1188 (2014). 24533501 10.1021/mp400610b

[39] Simón-Gracia L., Hunt H., Scodeller P., Gaitzsch J., Kotamraju V. R., Sugahara K. N., Tammik O., Ruoslahti E., Battaglia G., Teesalu T., iRGD peptide conjugation potentiates intraperitoneal tumor delivery of paclitaxel with polymersomes. Biomaterials 104, 247–257 (2016). 27472162 10.1016/j.biomaterials.2016.07.023 PMC5687559

[40] Battaglia G., Ryan A. J., Bilayers and interdigitation in block copolymer vesicles. J. Am. Chem. Soc. 127, 8757–8764 (2005). 15954782 10.1021/ja050742y

[41] Battaglia G., Ryan A. J., Tomas S., Polymeric vesicle permeability: A facile chemical assay. Langmuir 22, 4910–4913 (2006). 16700572 10.1021/la060354p

[42] Leson A., Filiz V., Förster S., Mayer C., Water permeation through block-copolymer vesicle membranes. Chem. Phys. Lett. 444, 268–272 (2007).

[43] Volpe G., Gigan S., Volpe G., Simulation of the active Brownian motion of a microswimmer. Am. J. Phys. 82, 659–664 (2014).

[44] James A. E., Driskell J. D., Monitoring gold nanoparticle conjugation and analysis of biomolecular binding with nanoparticle tracking analysis (NTA) and dynamic light scattering (DLS). Analyst 138, 1212–1218 (2013). 23304695 10.1039/c2an36467k

[45] Jang W.-S., Park S. C., Reed E. H., Dooley K. P., Wheeler S. F., Lee D., Hammer D. A., Enzymatically triggered rupture of polymersomes. Soft Matter 12, 1014–1020 (2016). 26616557 10.1039/c5sm01881a PMC5148629

[46] Tao Z., Raffel R. A., Souid A.-K., Goodisman J., Kinetic studies on enzyme-catalyzed reactions: Oxidation of glucose, decomposition of hydrogen peroxide and their combination. Biophys. J. 96, 2977–2988 (2009). 19348778 10.1016/j.bpj.2008.11.071 PMC2711289

[47] Bankar S. B., Bule M. V., Singhal R. S., Ananthanarayan L., Glucose oxidase—An overview. Biotechnol. Adv. 27, 489–501 (2009). 19374943 10.1016/j.biotechadv.2009.04.003

[48] Fullstone G., Wood J., Holcombe M., Battaglia G., Modelling the transport of nanoparticles under blood flow using an agent-based approach. Sci. Rep. 5, 10649 (2015). 26058969 10.1038/srep10649 PMC4462051

[49] Wright E. M., Loo D. D. F., Hirayama B. A., Biology of human sodium glucose transporters. Physiol. Rev. 91, 733–794 (2011). 21527736 10.1152/physrev.00055.2009

[50] Robinson K. L., Weaver J. V. M., Armes S. P., Marti E. D., Meldrum F. C., Synthesis of controlled-structure sulfate-based copolymers *via* atom transfer radical polymerisation and their use as crystal habit modifiers for BaSO_4_. J. Mater. Chem. 12, 890–896 (2002).

[51] Mantovani G., Lecolley F., Tao L., Haddleton D. M., Clerx J., Cornelissen J. J., Velonia K., Design and synthesis of *N*-maleimido-functionalized hydrophilic polymers via copper-mediated living radical polymerization: A suitable alternative to pegylation chemistry. J. Am. Chem. Soc. 127, 2966–2973 (2005). 15740133 10.1021/ja0430999

[52] Du J., Tang Y., Lewis A. L., Armes S. P., pH-sensitive vesicles based on a biocompatible zwitterionic diblock copolymer. J. Am. Chem. Soc. 127, 17982–17983 (2005). 16366531 10.1021/ja056514l

[53] Ryan A. J., Mai S.-M., Fairclough J. P. A., Hamley I. W., Booth C., Ordered melts of block copolymers of ethylene oxide and 1,2-butylene oxide. Phys. Chem. Chem. Phys. 3, 2961–2971 (2001).

[54] Müller-Plathe F., van Gunsteren W. F., Solvation of poly(vinyl alcohol) in water, ethanol and an equimolar water-ethanol mixture: Structure and dynamics studied by molecular dynamics simulation. Polymer 38, 2259–2268 (1997).

[55] Takasato Y., Rapoport S. I., Smith Q. R., An in situ brain perfusion technique to study cerebrovascular transport in the rat. Am. J. Physiol. Heart Circ. Physiol. 247, H484–H493 (1984). 10.1152/ajpheart.1984.247.3.H484 6476141

